# Luteolin-7-*O*-β-d-Glucoside Inhibits Cellular Energy Production Interacting with HEK2 in Keratinocytes

**DOI:** 10.3390/ijms20112689

**Published:** 2019-05-31

**Authors:** Ramona Palombo, Sabrina Caporali, Mattia Falconi, Federico Iacovelli, Blasco Morozzo Della Rocca, Alessandro Lo Surdo, Elena Campione, Eleonora Candi, Gerry Melino, Sergio Bernardini, Alessandro Terrinoni

**Affiliations:** 1 Department of Experimental Medicine, University of Rome Tor Vergata, Via Montpellier, 1, 00133 Rome, Italy; nemo26@hotmail.it (R.P.); alessandro.losurdo@gmail.com (A.L.S.); candi@uniroma2.it (E.C.); melino@uniroma2.it (G.M.); bernards@uniroma2.it (S.B.); 2 Laboratory of Cellular and Molecular Neurobiology, Fondazione Santa Lucia, Via del Fosso di Fiorano, 64, 00143 Rome, Italy; 3 Department of Industrial Engineering, University of Rome Tor Vergata, 00133 Rome, Italy; sabrynetta5@hotmail.it; 4 Department of Biology, University of Rome Tor Vergata, Via della Ricerca Scientifica, 00133 Rome, Italy; falconi@uniroma2.it (M.F.); federico.iacovelli@uniroma2.it (F.I.); blasco.morozzo.della.rocca@uniroma2.it (B.M.D.R.); 5 Department of Systems Medicine, Dermatologic Unit, University of Rome Tor Vergata, 00133 Rome, Italy; campioneelena@hotmail.com; 6 IDI-IRCCS, Biochemistry Laboratory, via dei Monti di Creta, 104, 00167 Rome, Italy

**Keywords:** luteolin-7-*O*-β-d-glucoside, glycolysis, hexokinase inhibitor

## Abstract

Flavonoids have been demonstrated to affect the activity of many mammalian enzyme systems. Their functional phenolic groups are able to mediate antioxidant effects by scavenging free radicals. Molecules of this class have been found able to modulate the activity of kinases, phospholipase A2, cyclooxygenases, lipoxygenase, glutathione S-transferase, and many others. Recently, it has been demonstrated that luteolin, in the form of Luteolin-7-*O*-β-d-glucoside (LUT-7G) is able to induce the keratinocyte differentiation process in vitro. This flavonoid is able to counteract the proliferative effects of IL-22/IL6 pathway by the inhibition of STAT3 activity also in vivo in a psoriatic mouse model. Observations on energy metabolism changes of differentiating cells led us to perform a complete metabolomics analysis using human primary keratinocytes treated with LUT-7G. Our results show that LUT-7G, is not only able to impair the nuclear translocation of STAT3, but it also blocks the energy metabolism pathway, depressing the glycolytic and Krebs pathway by the inhibition of hexokinase 2 activity. These data confirm that LUT-7G can be proposed as a potential candidate for the treatment of inflammatory and proliferative diseases, but its role as a hexokinase 2 (HEK2) inhibitor opens new perspectives in nutritional science, and especially in cancer therapy, in which the inhibition of the Warburg effect could be relevant.

## 1. Introduction

Recent literature data show interesting positive effects of plant-derived molecules in clinical trials regarding the treatment of inflammatory skin diseases [1,2], and a wide interest has been stimulated by flavonoids.

Flavonoids molecules (Figure S1) have been reported to have important effects in plant biochemistry. Recently, the main interest in these substances has been inspired by the antioxidant and anti-inflammatory activity of these polyphenolic compounds, leading to potential health benefits [3,4,5,6]. The presence of phenolic groups in these molecules mediates their antioxidant effects by its ability to scavenge free radicals and/or by chelating metal ions. Many flavonoids were found to be able to modulate the activity of different kinases, phospholipase A2, ciclooxygenases, lipoxygenase, glutathione S-transferase [4], aromatase, and many others [5]. Moreover, flavonoids have also been studied for their calcium homeostasis activities and as modulators of inflammation [7]. It has been demonstrated that the growth of T-lymphoid leukemia cells was inhibited by baicalein, by inhibition of protein tyrosine kinase (PTK), and by the induction of FS-7-associated surface antigen (FAS or CD95) mediated apoptosis [8]. Genistein induced apoptosis has also been shown in a subset of human thymocytes (CD32, CD41, CD81) by the inhibition of topoisomerase-II [9], and a variety of other examples are present in the literature (revised in [10]). 

Luteolin is mainly present in plants in the glycosylated form, with the glycoside hydrolyzed during absorption [11]. In our recent work [12] we demonstrated that luteolin, as luteolin-7-*O*-β-d-glucoside (LUT-7G) is able to induce keratinocyte differentiation process in vitro, counteracting the proliferative effects of critical mediators in psoriasis, like IL-22 and IL6 [13]. This proliferative pathway is achieved by enhancing the transcription of signal transducer and activator of transcription 3 (STAT3). LUT-7G has been shown to be able to inhibit the nuclear translocation of STAT3, also in a in vivo psoriasis Imiquimod (IMQ) mouse model [1,12,14]. However, the inhibition of STAT3 does not completely explain the activity of LUT-7G in keratinocyte differentiation. 

During the differentiation program, keratinocytes undergo a transformation in corneocytes, which implies a strong modification of cell structure and function [15,16], losing their main internal structures, such as the nucleus, endoplasmic reticulum (RE), and Golgi [17,18]. These changes involve, above all, metabolic pathways alteration. For this reason, a complete metabolomics analysis has been performed using human primary keratinocytes (HEKn) treated with LUT-7G, and the results have been compared to normal keratinocyte differentiation.

## 2. Results

### 2.1. Metabolic Analysis in LUT-7G Treated Keratinocytes

**Figure 1:**
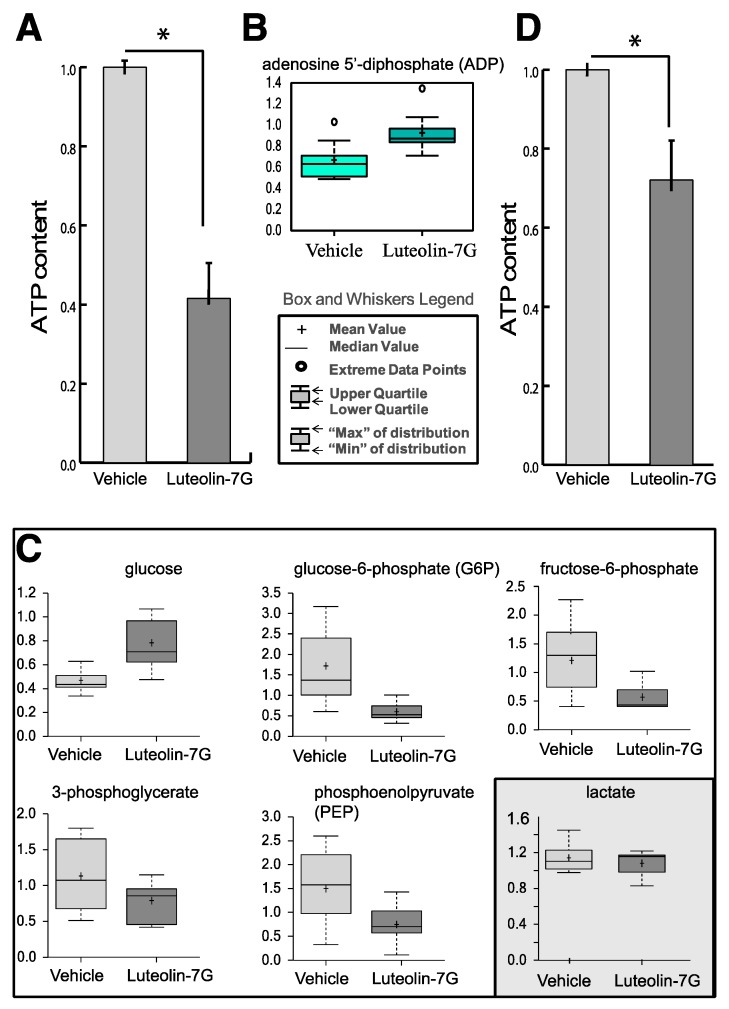
(**A**) Cellular ATP content in keratinocyte treated with LUT-7G showing a reduction of about 60%. (**B**) ADP content. (**C**) Metabolic analysis showing the decrease of G6P, F6P, and 3PG, as well of phosphoenolpyruvate (PEP). The cellular glucose (first upper panel) remains high inside cells. The data are represented as a boxplot from nine replicate samples. (**D**) ATP content of HaCaT cells (immortalized keratinocytes) showing a reduction of about 25% of ATP after LUT-7G treatment. * *p* < 0.05.

A complete metabolomic analysis has been performed using HEKn cells at passage P3. A 3 days of treatment with LUT-7G at a final concentration of 20 M in medium, was shown to be effective on keratinocyte differentiation [12]. The metabolic analysis was performed considering a data set platform of 279 metabolites (see Materials and Methods) [12]. The samples were loaded in an equivalent manner; each point with nine replicates of 10^6^ cells across the platform, and normalized for protein content prior to the analysis. Biochemical data are displayed as box plots and the legend is reported in Figure 1.

Interesting metabolic changes were detected in the major energy metabolism pathways. The total amount of ATP present in the cells was depressed of about 60% compared to the untreated control, and this indicates that a strong decrease of energy production in LUT-7G-treated keratinocytes occurred (Figure 1A). This is further confirmed by the increase of the bi-phosphorylated form of the nucleotide (ADP, Figure 1B). 

The measurement of the intermediate metabolites of the glycolysis pathway demonstrated a general depression (Figure 1C). In fact, the levels of glucose-6-phosphate (G6P) and fructose-6-phosphate (F6P), belonging to the first part of the pathway, in which the glucose was activated using an ATP molecule, were diminished with respect to the untreated control. The metabolites of the second part of the pathway, 3-phosphoglicerate (3PG) and phosphoenolpyruvate (PEP), were also diminished in comparison with the untreated control, indicating that the complete pathway was partially blocked by luteolin treatment (Figure 1C). Interestingly, glucose up-take by keratinocyte cells was not impaired by the flavone. In fact, higher levels of this metabolite were detected in LUT-7G treated cells, respect to the untreated controls, indicating that the glucose carriers were active and importing glucose inside cells. Thus, luteolin can act as a blocker of the glycolytic pathway but it does not affect the glucose supply, and is instead induced by the related compound apigenin [19].

Thus, the higher glucose content in LUT-7G treated cells could be justified by a decreased glucose use in the analyzed pathways. In line with this assumption, an increased ADP level was detected. It is interesting to note that the treatment had only little effects in HaCaT cells, demonstrating a different behavior between primary and immortalized cells (Figure 1D), that were not able to profoundly differentiate.

**Figure 2:**
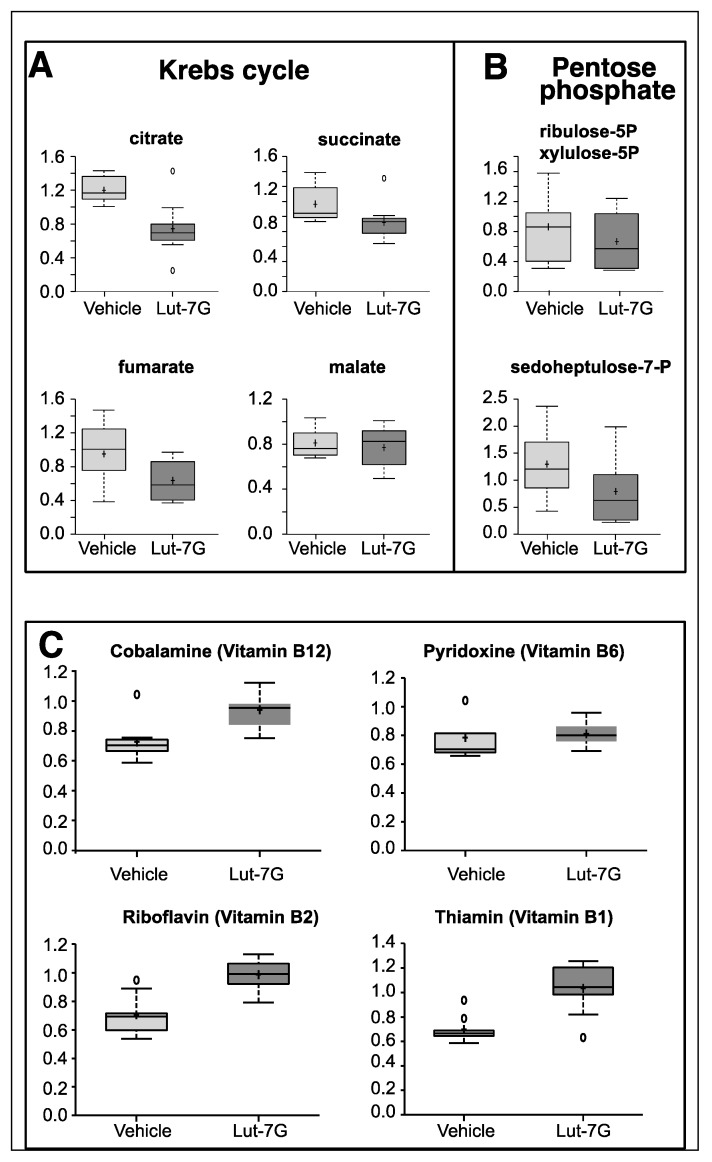
(**A**) Metabolomic analysis of tricarboxylic acid cycle or krebs cycle (TCA) intermediates showing the depression of citrate, succinate, fumarate, and malate in LUT-7G treated keratinocytes. (**B**) Analysis of intermediate of pentose phosphate pathway also shows a downregulation. (**C**) The analysis of important vitamin cofactors shows increased availability.

There are a number of distinct cellular processes able to produce ATP; the three main pathways that can be involved are glycolysis, tricarboxylic acid cycle or krebs cycle (TCA), and the pentose phosphate pathway (PPP). The analysis of metabolites of TCA cycle, like citrate, succinate, and fumarate demonstrated that this metabolic pathway was also impaired since all the intermediates were strongly reduced in the treated cells (Figure 2A). The analysis of PPP also showed a reduction of the intermediate metabolites like sedoheptulose-7-P and xylulose-5P (Figure 2B). 

Many enzymatic cofactors of the energy metabolism pathway were also analyzed, in particular those that follow: Vitamin B6 (pyridoxal 5’-phosphate), a required coenzyme of glycogen phosphorylase (Figure 2C), cobalamin (vitamin B12), involved in the metabolism of the proprionyl-CoA and in the metabolism of amino acids, riboflavin (vitamin B2), a central component of the cofactors flavin adenine dinucleotide (FAD) and flavin mononucleotide (FMN) that function as cofactors for a variety of flavoprotein enzyme reactions, many of which are important in the electron transport chain and in decarboxylation of pyruvate and ketoglutarate, thiamine pyrophosphate (TPP), which represents a coenzyme in the catabolism of sugars and amino acids. As reported in Figure 2C, higher levels of these molecules were present in LUT-7G treated cells. This confirms a reduced consumption of these molecules as part of the glycolytic pathway that was found to be impaired in the treated cells.

### 2.2. Metabolic Analysis in Calcium Differentiating Keratinocytes

Treatment with LUT-7G was shown to induce differentiation in keratinocytes [12]. To understand the relationship between the inhibition of energy production and keratinocytes differentiation, we induced these cells to differentiate by culturing in a medium containing 1.2 mM calcium, which is well known to promote keratinocyte differentiation in vitro [20,21]. 

**Figure 3:**
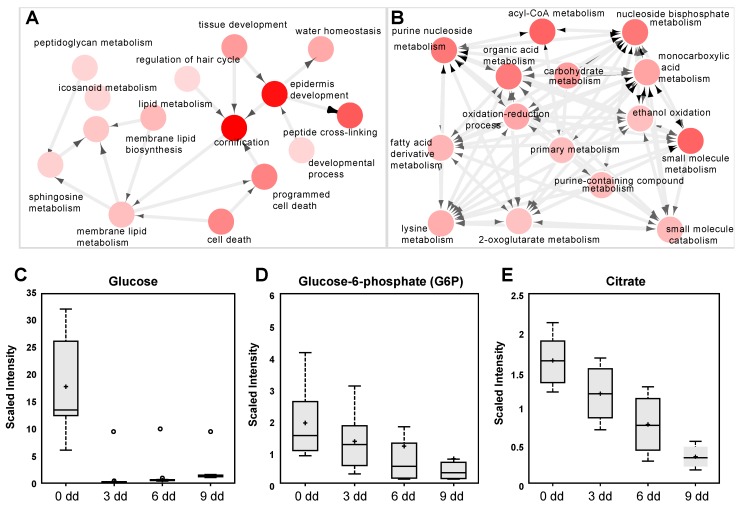
(**A**) Clustering and annotation analysis (methods) from RNAseq experiment in differentiated keratinocytes. The nodes are the processes where most regulated gene cluster, the arrows represent the connections between the processes. As is visible, there is a preponderant association with epidermis differentiation, cornification, and peptide crosslinking. (**B**) The same analysis showed another cluster (of downregulated genes) acknowledging downregulation of energy pathway metabolism. (**C**–**E**) Analysis of metabolic intermediate of glycolysis and Krebs cycle in differentiating keratinocytes, showing depression of G6P and citrate. In this case (first panel), a strong decrease of cellular content of glucose is visible.

The differentiated keratinocytes (6 days of calcium treatment) were analyzed by RNAseq [22], using Gorilla and Cytoscape software packages for gene annotation and clustering. The analysis showed a clustering of keratinocyte differentiation markers in a characteristic pattern, involving “Cornification (GO:0070268)”, “Epidermis development (GO:0008544)”, and “Peptide cross-linking (GO:0018149)”. The clustering using Cytoscape is reported in Figure 3A, where the processes are represented as nodes (circles), while arrows represent the connection between different processes. These data demonstrate that the differentiation program in these cells was achieved, as indicated by the increase of TG1, TG3, K10, loricrin, involucrin [17,18,23,24], and the strong decrease of p63, the principal mediator of epidermal homeostasis and differentiation. A summary of involved genes is presented in Table S1. Processes governing cell metabolism that mainly contain downregulated genes, represent another interesting clustering. As shown in Figure 3B, the processes involve “acyl-CoA metabolic process (GO:0006637)”, “carboxylic acid metabolic process (GO:0019752)”, “ribonucleoside bisphosphate metabolic process (GO:0033875)”, “acetyl-CoA metabolic process (GO:0006084)”, and others, with interesting genes summarized in Tables S1 and S2. To comparatively investigate the effect of the flavonoid in cell energy metabolism we performed a metabolic analysis in calcium-differentiated keratinocytes with the same techniques used for LUT-7G. The results demonstrated that there was an inhibition of the energy production also during calcium differentiation, as shown by the decrease of G6P and citrate (Figure 3B,C). Both glycolysis and Krebs cycle seem to be involved, as in luteolin experiments. These results show that the inhibition of energy production from glucose is an important step of epidermal keratinocyte differentiation.

However, the mechanism by which this inhibition was achieved seems to be different. As shown in Figure 3C, in Ca-induced keratinocytes, the internal glucose level rapidly decreased, while in LUT-7G treated cells it remained high (Figure 1C).

### 2.3. The Role of LUT-7G in the Regulation of Hexokinase 2 Activity

**Figure 4:**
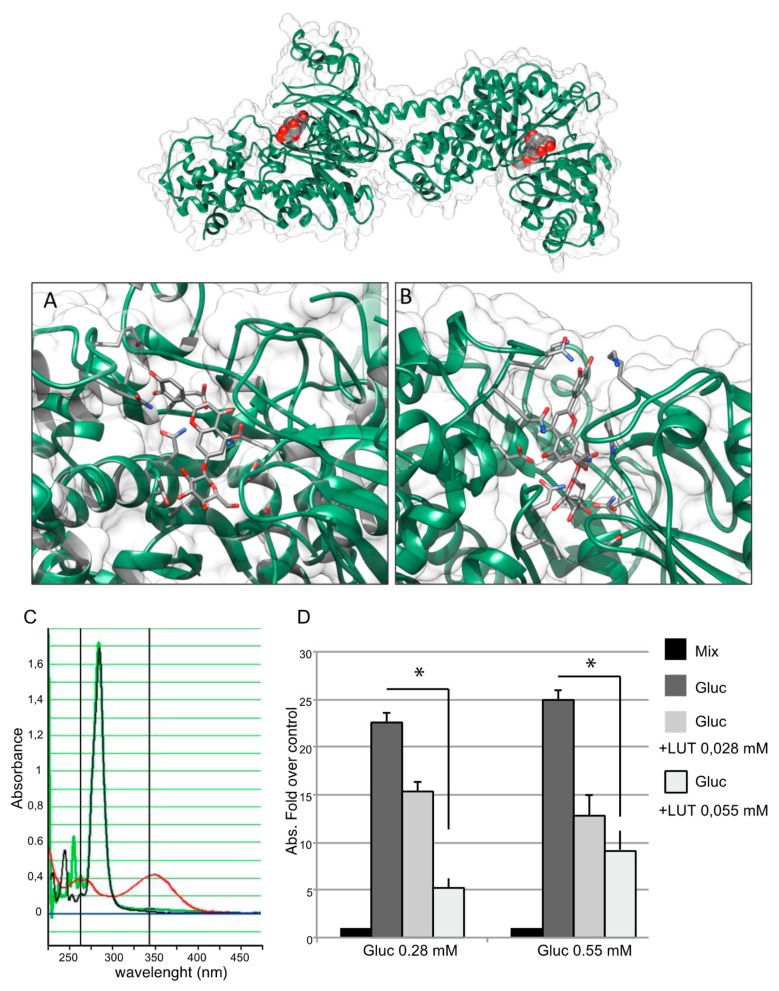
Top, best docking complexes between HEK2 and LUT-7G. The luteolin molecules, hosted in the active sites of HEK2, are depicted by spacefill representations colored by atom type, while HEK2 is shown as a green cartoon. (**A**) Site 1 and (**B**) site 2, detailed views of the best docking solutions. The LUT-7G molecules are depicted by stick representations, while the protein is shown as a green cartoon. The molecular views have been produced by using the program UCSF Chimera [25]. (**C**) Spectrophotometric analysis for NADH measuring. The sample containing the reaction mixture without glucose is indicated by a black curve, the sample containing LUT-7G shows a characteristic absorbance curve of luteolin with a peak around 350 nm (red curve). When the LUT-7G was added to the reaction mixture (green line) the absorbance peaks disappeared. (**D**) The presence of NADH was evaluated measuring absorbance at 340 nm. The data are expressed as fold over control (absorbance of reaction mix), in the left group a concentration of glucose of 0.28 mM was used, and two concentrations of LUT-7G of 0.028 and 0.055 mM, respectively. In the right-hand group of columns, a concentration of glucose of 0.55 mM was used. In both experiments a reduction of NADH synthesis is visible. *t*-test, * *p* < 0.05.

To detect a direct effect of LUT-7G leading to the inhibition of the first glucose-consuming enzyme, protein-ligand molecular docking was used to predict the complexes between the human hexokinase 2 enzyme with LUT-7G (see Materials and Methods for details). In the first step, the entire surface of the molecule was sampled and LUT-7G was found to preferentially bind in the two catalytic sites previously occupied by the crystallized inhibitor. These initial poses showed a negative energy value of about −8.0 kcal/mol (Figure 4 top). 

**Figure 5:**
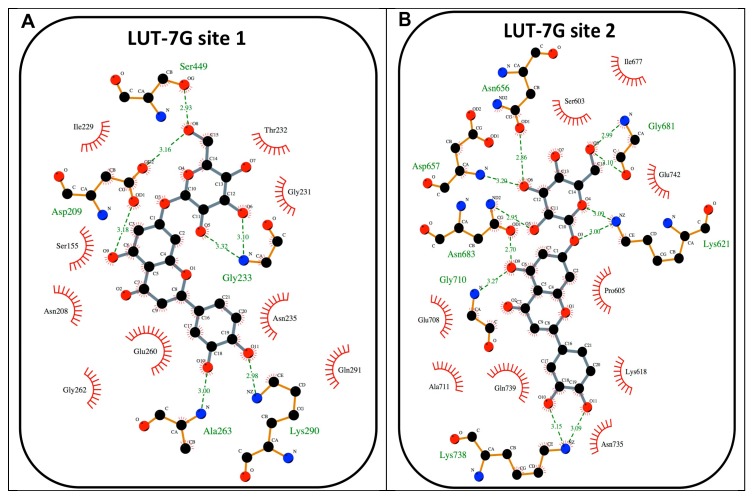
(**A**) Schematic view of the best HEK2 active site docking solution with LUT-7G for site 1. The 2D depiction shows hydrogen bonds as green dashed lines between the interaction partners. The residues that are in proximity of the ligand are indicated. This image was produced using the LigPlot+ software [26]. (**B**) A schematic view of the best HEK2 active site docking solution with LUT-7G for site 2.

**Figure 6:**
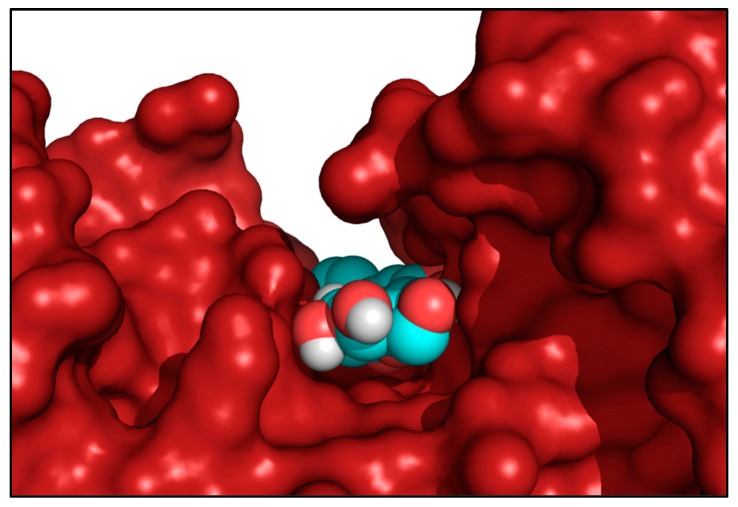
Side view of HEK2 active site 1 showing the bound LUT-7G molecule located by the docking procedure at the bottom of the active site crevice. The spacefill representation colored by atom type indicates the LUT-7G molecule, while the enzyme was rendered as a red solid surface. This picture was produced by using the program PyMol [27].

Subsequently, through a localized search focusing on the HEK2 binding sites, LUT-7G binding energies were improved. These second docking simulations further confirm the high affinity of LUT-7G for these sites, indicating greater interaction energy values of -9.6 and -11.9 kcal/mol for site 1 and 2, respectively (Figure 4A,B and Figure 5A,B). As a comparison, the docking energies evaluated for ATP were lower, reaching the values of –8.7 and –10.0 kcal/mol for site 1 and 2, respectively (Figure S2). Interestingly, when the C-2-substituted glucosamine inhibitor was re-docked in the HEK2 active sites it attained a docking energy of –7.4 and –10.3 kcal/mol, for site 1 and 2, respectively, indicating a greater affinity of LUT-7G. The docking simulations accommodated these molecules at the bottom of the active site crevices, almost completely obstructing substrate access (Figure 6). In particular, both the glucoside and the luteolin moieties were fully stabilized by several hydrogen bonds and hydrophobic contacts established with the residues shown in Figure 5A,B. Both ATP and C-2-substituted glucosamine inhibitor showed lower affinities for the sites (Figure S2), suggesting that the LUT-7G molecule represented a strong HEK2 inhibitor. Although in HEK2 the two binding sites were different (i.e., in residue composition and three-dimensional structure), the ligands energies and poses observed were quite similar.

To investigate the inhibition potential of LUT-7G on HEK2, we used a coupled enzyme system in which the glucose, firstly converted by the enzyme in G6P, was transformed in 6-phosphogluconate with a further reaction catalyzed by G6PDH, with the release of NADH, that was spectrophotometrically measured at 340 nm (see Materials and Methods section). However, the use of this system was limited due to LUT-7G absorption at a similar wavelength with a peak at 353 nm. We conducted an initial experiment to evaluate the absorbance of LUT-7G alone and the mixture containing the enzyme, using a concentration of 0.055 mM of LUT-7G, since higher concentrations saturate the absorbance between 320 and 375 nm. The results showed no absorbance in samples containing the buffer with the enzyme mix – from 260 nm to all visible spectra (Figure 4C, black line). In the sample containing only LUT-7G, a characteristic absorbance curve of the luteolin is visible, with a peak around 350 nm (Figure 4C, red line). Interestingly, when LUT-7G was added to the reaction mixture (no glucose) the absorbance peak disappeared (Figure 4C, green line). This disappearance was probably due to a shift in wavelength, indicating an interaction with the enzyme. To avoid overlap between LUT-7G and NADH absorption, we tested the absorbance reached with two concentrations of glucose and the effect of LUT-7G used with the dilutions specified in the Materials and Methods and in Figure 4. The result showed a reduction of NADH synthesis when LUT-7G was added to the mix containing glucose (Figure 4D, left group of columns), with a proportional reduction of NADH when the concentration of luteolin was doubled. When using the higher concentration of glucose, a similar effect was visible. 

The disappearance of the absorption peak (Figure 4C, green line) of LUT-7G in the presence of the enzyme could be explained by its particular conformation when bound. Indeed, as observed in both the HEK2 sites by docking, luteolin was constrained in a conformation in which its main rings displayed a torsion of about 60° from the coplanar conformation (Figure 4A,B). As previously reported, the absorption of LUT-7G in solution refers to transition between molecular orbitals that are spread on all aromatic rings in a planar orientation [28,29]. The mutual rings orientation induced by binding may disrupt the orbitals, thus explaining the observed loss of absorption peak (Figure 4A,B). 

Even if equal concentrations of glucose and LUT-7G could not be tested in the spectrophotometric system due to the absorbance band overlap, (0.55 mM of LUT-7G produce an absorbance value of 2.0) all the data are indicative of a potential inhibitory effect of LUT-7G on HEK2 enzyme.

## 3. Discussion

One of the key genes of epidermal development and regulation of keratinocytes differentiation is the short p63 isoform, ΔNp63, which has been confirmed to be reduced in our differentiation experiment (Table S1). It plays a central role in controlling the differentiation/proliferation of basal keratinocytes [16,30], by regulating the expression of epidermal specific genes [31,32,33]. Recently, in epithelial cells, it has been demonstrated, that p63 is also a regulator of cellular energy metabolism and respiration [34]. In fact, the ΔNp63 downregulation leads to the reduction of oxygen consumption and to the lowering of the energetic request through the glycolysis pathway.

ΔNp63 depletion is essential in keratinocyte differentiation. In the higher epidermal layer this transcription factor decreases its expression, and RNAi experiments have demonstrated that the artificial inhibition of ΔNp63 is sufficient to induce keratinocyte differentiation [31,35]. Accordingly, we showed that during keratinocyte differentiation there was a depression of the glucose metabolism (Figure 3C,D). Moreover, ΔNp63 is able to control the insulin receptor substrate (IRS) IRS1 [36], and in our p63RNAi experiments, it also controls IRS4 (Table S1, last row), both of which are responsible of the re-localization of GLUT4 transporter [37] on the plasma membrane, explaining the mechanism of glucose uptake impairment observed during differentiation.

This leads us to hypothesize that two different mechanisms are present in inducing keratinocyte differentiation. One in which LUT-7G acts in the first regulatory step of glycolysis, on HEK2, avoiding the phosphorylation of glucose in G6P and leading to the persistence of unused glucose inside cells. The second mechanism, relative to the normal differentiation, in which the continuous uptake of glucose by the external membrane space could be impaired by the reduction of p63 expression, affecting the transporters mediated uptake of glucose. This leads to the consumption of the internal reserve of glucose with the subsequent reduction of glycolytic production of ATP and pyruvate.

Our data show that LUT-7G, is not only able to impair the nuclear translocation of STAT3 [12] inhibiting keratinocyte proliferation and inducing differentiation, but the latter phenomenon is also synergistically achieved by blocking the energy metabolism pathway. Indeed, the reduction of the energetic demand through the depression of the glycolytic and Krebs pathways is a key regulator of keratinocyte differentiation. This is achieved by LUT-7G with the inhibition of HEK2 activity, via the binding of the compound in the active sites of the enzyme with an energy similar to a potent HEK2 inhibitor [38].

These results expand the biochemical properties of this flavonoid, leading us to consider its differentiative and cytostatic properties as the sum of the action on different cellular pathways. We now can expand LUT-7G action on the inhibition of cellular energy metabolism pathway, by the interaction with HEK2. There are a number of publications indicating an anticancer activity of this flavonoid, in which cell cycle arrest [39] was described by the increase of Bax/ Bcl-XL ratio mediated by depression of p21; and also the induction of endoplasmic reticulum (ER) stress [40] was shown. Luteolin has been also shown to sensitize colon cancer cells to apoptosis induced by TNF, through the suppression of NF-kappaB [41]. This activity has been supposed to reside in the modulation of reactive oxygen species (ROS). However, a very interesting biochemical phenomenon occurring in cancer cells is the Warburg effect [42]. Cancer cells show an altered metabolism – they increase the glucose uptake and the transformation of glucose to lactate in the presence of oxygen. This phenomenon is observed even in the presence of intact and functioning mitochondria [43,44]. Furthermore, the PI3K-Akt-mTOR pathway coordinates the uptake and use of multiple nutrients, such as glucose, glutamine, nucleotides, and lipids, enabling growth and proliferation of cancer cells. Especially in solid cancers, preclinical tests have shown that the use of PI3K or mTOR inhibitors results in the restoration of sensitivity of cancer to therapy. It has also been already demonstrated that luteolin can inhibit this signaling pathway impairing PI3K activity [20], suppressing Akt phosphorylation [41]. Today, specific inhibitory molecules have been generated with the aim of inhibiting this specific signaling pathway [45]. Furthermore, a wide variety of kinase inhibitors have been developed for cancer therapy. The human genome contains more than 500 different kinases, and they represent the second group of drug targets, after G-protein-coupled receptors, with more than 150 kinase targeted drugs in currently in clinical trials. (revised in [46]). 

## 4. Materials and Methods

### 4.1. Molecular Docking Simulations

Protein–ligand molecular docking represents a simulative technique able to predict the binding mode of a ligand on a receptor, taking into account both geometrical and electrostatic contributions. This computational method was used to predict the Luteolin 7-*O*-β-d-glucoside binding sites on HEK2.

The molecular docking simulations were executed using the AutoDock Vina 1.1.2 program [47], through the AutoDock/Vina PyMOL plugin (http://wwwuser.gwdg.de/~dseelig/adplugin.html) (The PyMOL Molecular Graphics System Version 1.5.0.4. Schrödinger, LLC; [48]).

The X-ray structure of human HEK2 complexed with the C-2-substituted glucosamine inhibitor (PDB ID: 5HG1) [38] was used as a receptor and the coordinates of the inhibitor and water were eliminated from the structure. In the first round of simulations, the docking box was chosen to include the whole enzyme (dimensions: 93.75 × 93.75 × 150.0 Å), to evaluate the presence of possible binding sites over the entire protein surface. In a second round of simulations, smaller docking boxes were used around both binding sites (dimensions: 34.98 × 34.98 × 56.25 Å each). Nine side chains in the first HEK2 ATP-binding site (Asn208, Asn235, Asp209, Gln291, Glu260, Glu294, Ile229, Lys290, Ser234), and fourteen side chains in the second site (Asn656, Asn683, Asn735, Asp657, Gln739, Glu708, Glu742, Lys618, Lys621, Lys738, Phe604, Ser603, Ser682, Thr658), were considered rotatable to improve flexibility of the receptor during the docking simulations. The VINA exhaustiveness parameter was set to 25 to increase the local minimum finding probability. The docking simulations were carried out for the luteolin and the ATP to evaluate their relative affinities. The Luteolin 7-*O*-β-d-glucoside (PubChem CID: 5280637) and the ATP (PubChem CID: 5957) coordinates were downloaded as SDF from the PubChem compound database (https://pubchem.ncbi.nlm.nih.gov) and converted in PDB files using the PyMol program [27]. The molecular pictures have been produced by using the programs PyMol [46] and UCSF Chimera [25].

### 4.2. ATP Levels Detection

The cells were cultured and treated for 3 days with Lut-7G, then harvested, counted, and analyzed by bioluminescence. The ATP level was measured based on a luciferin–luciferase reaction, using the ADP/ATP Ratio Assay Kit (Abcam, Cambridge, UK,), following the manufacturer’s instructions. See also [49] for more information on this.

### 4.3. Cell culture and Treatments

Human neonatal epidermal keratinocytes (HEKn) (Invitrogen, Carlsbad, CA, USA,) were cultured using Epilife medium including human keratinocyte growth supplements (Cascade). Cells were plated in collagen-coated dishes, to keep the cells subconfluent avoiding triggering of differentiation. At each time point the cells were harvested, counted for RNA and protein extraction. 

Cell differentiation was induced by adding 1.2 mM CaCl2 to the medium for different time periods (2, 3, or 6 d).

Luteolin 7-glucoside (Sigma Aldrich, St. Louis, MI, USA) was dissolved in DMSO, and stored at +4 °C kept protected from light. It was used at a final concentration of 0.01% *v*/*v* in media. 

### 4.4. Metabolomic Analysis 

Cells were collected at passage 3 (P3) after 3 days of treatment. The control cells were grown with the vehicle used to dissolve the flavone (DMSO at final concentration in medium of 0.01% *v*/*v*), treated cells were grown with the addition of luteolin 7-glucoside 20 µM in medium. For each condition, 9 replicates were obtained, with 10^6^ cells each. The results presented are all significant with *p* < 0.05.

Collected samples were stored at −80 °C, extracted and prepared using a standard metabolic solvent extraction method (see [12] for details).

### 4.5. Hexokinase Activity

For hexokinase 2 activity tests we used the Glucose Assay GAHK20-1KT (Sigma Aldrich). According to the manufacturer, the standard concentration of glucose is 5.5 mM, but we used a final concentration of glucose of 0.550 and 0.275 mM (halved) for NADH measurement. In the first experiment, to evaluate the absorbance of the LUT-7G alone, a concentration of 0.055 mM was used, since higher concentrations saturate the absorbance between 320 and 375 nm.

## 5. Conclusions

In this paper, we highlight the effect of LUT-7G as HEK2 inhibitor leading to the downregulation of glycolysis. This regulatory mechanism, and its action as a specific inhibitor of HEK2, opens the possibility of further investigations regarding the role of this flavonoid molecule in cancer biology, and not only in the treatment of inflammatory and proliferative diseases, as previously supposed.

## Supplementary Materials

Supplementary materials can be found at https://www.mdpi.com/1422-0067/20/11/2689/s1.

## Abbreviations

LUT-7G	luteolin-7glucoside
HEK2	hexokinase 2
GLUT-4	glucose transporter type 4
TCA	tricarboxylic Acid Cycle or Krebs cycle
PEP	phosphoenolpyruvate
3PG	3-phosphoglycerate
G6P	glucose-6-phosphate
IMQ	imiquimod
IL-22	interleukin-22
IL6	interleukin-6
STAT3	transcription of Signal transducer and activator of transcription 3
HEKn	human epidermal keratinocytes neonatal
TG1, TG3	transglutaminase 1 and 3
K10	keratin 10
