# Supplementary material for: Luteolin-7-O-β-d-Glucoside Inhibits Cellular Energy Production Interacting with HEK2 in Keratinocytes
Source: Int J Mol Sci. 2019 May 31;20(11):2689. doi: 10.3390/ijms20112689 (PMC6600217; doi:10.3390/ijms20112689)
Supplement: Supplementary file 1 [file ijms-20-02689-s001.zip › ijms-462812 suppl final/ijms-462812 suppl. figure.docx]

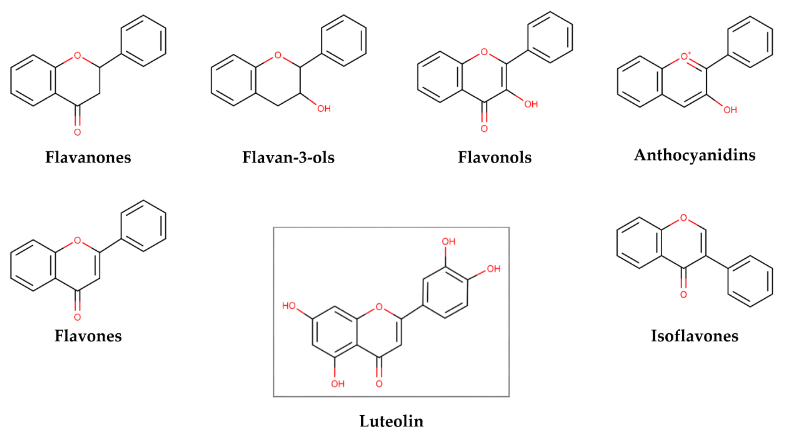


**Figure S1.** Chemical structures of flavonoid subclasses. The luteolin structure, belonging to the flabones subclass, is enclosed in a grey box. This picture has been produced using the program MarvinSketch version 19.7.0, <https://chemaxon.com/products/marvin>, 2019.

**
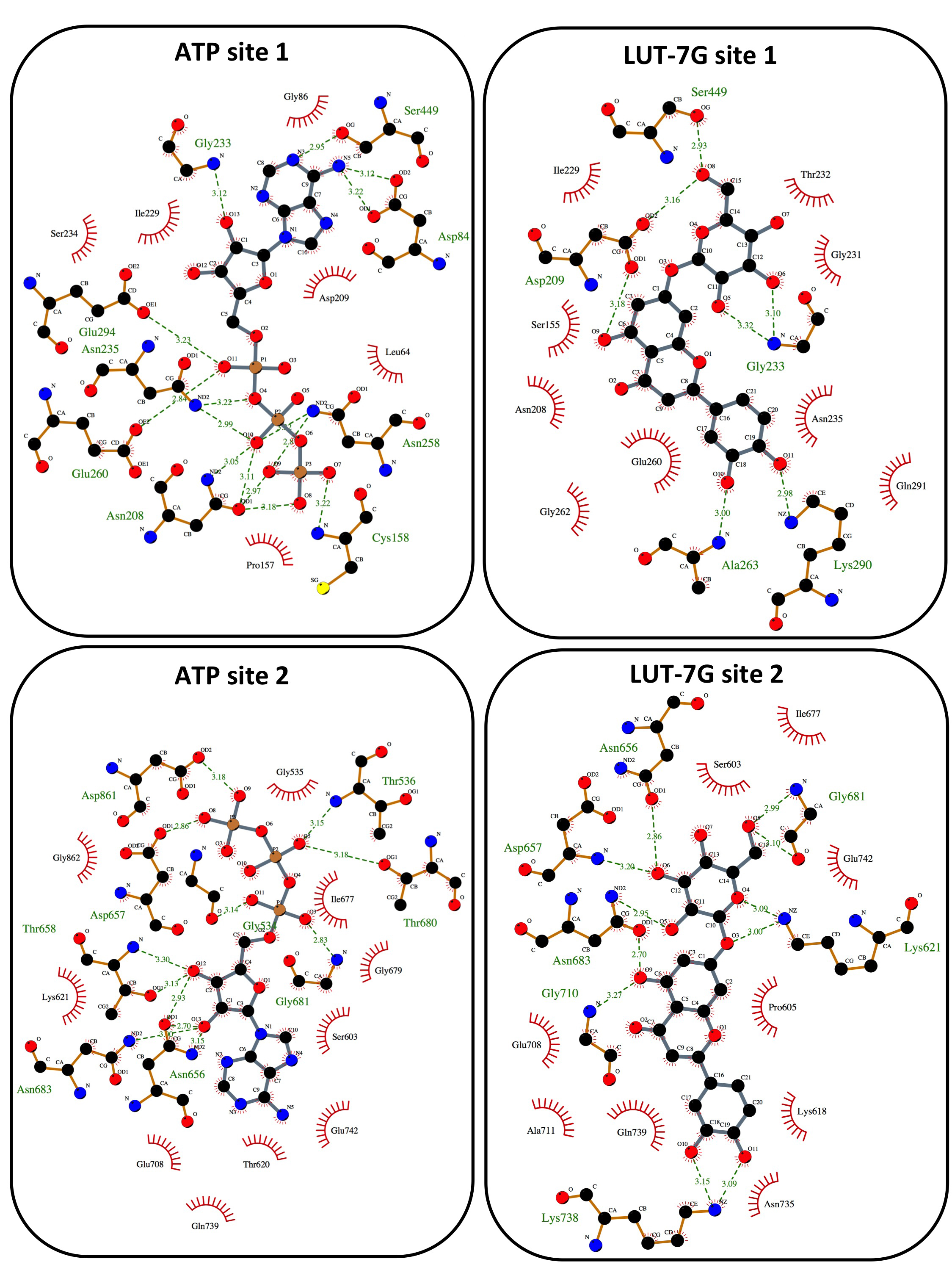
**

**Figure 2.** Schematic view of the best HEK2 active sites docking solutions with ATP (left panels), and LUT-7G (right panels). The 2D depictions show hydrogen bonds as green dashed lines between the interaction partners. The residues that are in proximity of the ligands are indicated. This image has been produced by the authors using the LigPlot+ software.
